# Kajal (Kohl) – A dangerous cosmetic

**DOI:** 10.4103/0974-620X.64242

**Published:** 2010

**Authors:** Anup Mohta

**Affiliations:** Department of Pediatric surgery, Chacha Nehru Bal Chikitsalaya, Delhi, India

Sir,

Application of ‘Kajal’ (also known as kohl or surma) is a common practice in Indian families. Although concerns about its safety have been raised, its use in pediatric age is very prevalent. The beliefs and practices with respect to use of ‘Kajal’ in children attending a tertiary care pediatric hospital in northern India are described.

One hundred consecutive children under 12 years of age of both genders and all religions (65% Hindus, 30% Muslims, and 5% other religions) were examined for use of ‘Kajal’. Eighty-six children had been applied ’Kajal’. Of these, 48 were girls and 38 were boys. Most of the children were under five years of age. On interview, the following information was collected.

- 64% mothers were educated up to class 12^th^ and more;
- 90% mothers applied ‘Kajal’ simply due to the advise of their elders;
- More than 50% parents did not know the advantage of applying ‘Kajal’. Some reasons cited included that it a) increases the size of eyes; b) improves eye sight; and c) protects the eyes against diseases.
- No mother who applied ‘Kajal’ could enumerate any disadvantage of using Kajal.
- About 80% respondents used ‘Kajal’ made at home. Most commonly, soot of the flame of an oil based lamp was mixed with some oil or eye ointment. Rest of the parents used commercially available ‘Kajal’.
- Of mothers not using ‘Kajal’, most did not have any reason for not using it while some said it was harmful to eyesight.

‘Kajal’ (Kohl) is a popular eye care product and its use has been reported since ancient times. Kohl (surma) has been defined as an eye preparation in ultra fine form of specially processed “Kohl Stone” (galena) incorporated with some other therapeutically active ingredients.[1] It has been claimed to keep the eyes cool and clean, improve vision and strengthen the eyes. It has also been used for the prevention and treatment of eye diseases such as blepharitis, cataract, conjunctivitis etc.[2] It is also said to ward off an ‘evil eye’.

Most commercially produced ‘kajal’ contain high levels of lead. Studies have revealed that ‘Kajal’ comprises of galena (PbS), minium (Pb_3_O_4_), amorphous carbon, magnetite (Fe_3_O_4_), and zincite (ZnO).[3] Prolonged application may cause excessive lead storage in the body, affecting the brain and bone marrow, causing convulsions and anemia. Dirty fingers, sharp and uneven fingernails of the caregivers are potentially harmful to the child’s eyes.[4] However, one study has questioned the authenticity of such reports and has claimed that ‘Kajal’ is safe for use.[5] US FDA does not permit its use in a cosmetic or in any other FDA-regulated product.[6]

Despite recommendations against the use of ‘Kajal’, it is routinely used in children. It is necessary that the health care providers should educate the parents regarding the use of Kajal. The steps that can be taken by different agencies can be

- Scientific bodies of pediatricians, ophthalmologists and pediatric surgeons should interact with social leaders for education of public.
- Parent education by pediatricians, ophthalmologists and other primary health care providers.
- Display of educative material in the health care facilities like hospitals and clinics.
- Regulatory directions to manufacturers regarding compulsory testing of ‘Kajal’ for levels of lead and other heavy metals and certification.
- Banning of advertisements proclaiming unauthenticated advantages of ’Kajal’ in media.
